# Relationships of affective temperament ratings to diagnosis and morbidity measures in major affective disorders

**DOI:** 10.1192/j.eurpsy.2021.2252

**Published:** 2021-11-23

**Authors:** Alessandro Miola, Ross J. Baldessarini, Marco Pinna, Leonardo Tondo

**Affiliations:** 1 Department of Neuroscience (DNS), University of Padova, Padua, Italy; 2 International Consortium for Mood & Psychotic Disorders Research, McLean Hospital, Belmont, Massachusetts, USA; 3 Department of Psychiatry, Harvard Medical School, Boston, Massachusetts, USA; 4 Lucio Bini Mood Disorders Centers, Cagliari, Italy

**Keywords:** Affective temperaments, bipolar disorders, diagnosis, major depression, TEMPS-A

## Abstract

**Background:**

Ratings of affective temperament types show promise in helping to differentiate diagnostic groups among major affective disorders as well as to predict associations with important aspects of morbidity including suicidal risk.

**Methods:**

The Temperament Evaluation of Memphis, Pisa, Paris, and San Diego auto-rating (TEMPS-A) questionnaire was completed by 858 unselected, consecutive, consenting adults diagnosed with a DSM-5 major affective disorder (173 bipolar-1 [BD-1]), 250 BD-2, 435 major depressive disorder [MDD]) to score for anxious (*anx*), cyclothymic (*cyc*), dysthymic (*dys*), hyperthymic (*hyp*), and irritable (*irr*) affective temperaments. We tested their associations with diagnosis and selected clinical factors, including diagnosis, depression scores, suicidal ideation or acts, substance abuse, episodes/year, and %-time ill.

**Results:**

Scores for *cyc* ranked: BD-2 > BD-1 > MDD; *anx* ranked: MDD > BD-2 > BD-1; *irr* was greater in BD than MDD; *dys* was greater in MDD than BD; *hyp* did not differ by diagnosis. We confirmed associations of suicidal risk with higher scores of all temperament types except lower *hyp* scores. Higher *cyc* and *irr* scores and lower *anx* scores were associated with substance abuse. Several scores were higher with measures of greater affective morbidity: *cyc* with current depression, episodes/year, and %-time ill; *irr* with more episodes and depressions/year and greater %-time manic. Some of these associations were selective for BD or MDD.

**Conclusions:**

The findings indicate that TEMPS-A ratings of affective temperament types can contribute to differential diagnoses and predict types and amounts of affective morbidity, as well as detecting suicidal risks.

## Introduction

Much more timely differentiation of major depressive (MDD), bipolar-1 (BD-1), and bipolar-2 (BD-2) disorders is clinically crucial to improving long-term planning aimed at better care of mood disorder patients [1]. Notably, the latency from initial clinical manifestations to a firm diagnosis and appropriate treatment of BD as distinct from unipolar depression averages 5–10 years, and even longer with onset in juvenile years, owing in large part to an excess of depression early in the course of BD [2,3]. In striking contrast, nearly half of lifetime risk of suicidal acts (attempts and suicides) occurs within the first 2–3 years of these illnesses, and indeed such acts make early diagnosis more likely [4]. The uncertain differentiation of BD from MDD is underscored by the finding that more than half of patients originally diagnosed with a depressive episode eventually meet diagnostic criteria for BD, often owing to missing diagnosis of BD-2 through failure to recognize hypomania [5]. In addition, prolonged duration of untreated illness in BD is associated with more suicide attempts, greater affective and behavioral instability, and possibly more prolonged future illness [6,7].

A potential contribution to improving early recognition of BD and MDD might include use of ratings of affective temperament or assessment of other aspects of temperament and personality, seeking potential links between a biological disposition to mood disorder and its clinical manifestations and considering some temperaments as antecedents of particular mood disorders [8,9]. Such assessments often rely on questionnaire-based rating schemes, including the Temperament Evaluation of Memphis, Pisa, Paris, and San Diego (TEMPS, often as a self- or auto-rating, TEMPS-A) [10], the Temperament and Character Inventory (TCI) [11], and the Neuroticism, Extraversion, Openness, Agreeableness, and Conscientiousness Scale (NEO-PI-3) [12]. In addition to supporting earlier, accurate diagnosis, such assessments might also have predictive value for the types and relative amounts of particular psychopathological features [13], and temperaments have shown strong relationships with suicidal behavior or ideation [14].

Several studies have focused on temperament assessments in individuals diagnosed with MDD [15–17] or with BD specifically [18–24], or have considered mood disorders together using TCI [25,26] or TEMPS [1,27–30]. Such studies have revealed differences between mood disorder patients and controls, including unaffected family members, with suggestive differences between BD and MDD patients and possibly between BD-1 and BD-2 patients based on ratings of cyclothymic, hyperthymic, and irritable temperaments, in particular, as well as ratings of harm-avoidance [1,31–33]. There are also preliminary suggestions that temperament assessments may help to predict responses to antidepressant or mood-stabilizing treatments [28].

However, most studies focusing on assessment of temperament in individuals with major mood disorders suffer from various limitations. These include small sample size, data not systematically analyzed by multivariate analyses, as well as retrospective or cross-sectional study designs that limit ability to form hypotheses regarding causality. In addition, it is important to consider potential effects of current mental state, which may influence responses to questions aimed at evaluating affective temperament [34]. Finally, relationships among affective temperament, morbidity indices, and clinical course in mood disorder patients have not been investigated extensively.

Given this background, the aim of the present study was to compare temperament profiles, assessed with the TEMPS-A questionnaire, in BD-1, BD-2 and MDD patient-subjects, and to test whether such assessments can contribute to differentiating among these diagnoses and can provide predictive associations with types or amounts of selected aspects of psychopathology.

## Methods

### Study subjects

Participants were adults evaluated and followed by the same mood disorder expert (LT) for several years at the Lucio Bini Mood Disorders Center in Cagliari, Sardinia, a specialized, academic outpatient clinic for the diagnosis, treatment, and study of affective disorder patients. They were consecutive and unselected except for adult age, presence of a DSM-5 major affective disorder (BD-1 or BD-2, MDD), and having completed TEMPS-A assessment. All were treated clinically and followed prospectively and systematically over several years. Written, informed consent was provided by all participants for collection and analysis of clinical data to be presented anonymously in aggregate form, following procedures approved by a local ethical review committee in accordance with requirements of Italian law and with the Helsinki Declaration. Study data were collected and entered a computerized database in coded form.

### Measures

Current and lifetime diagnosis, course of illness, and psychiatric comorbidities were assessed according to DSM-5 diagnostic criteria [35]. TEMPS-A assessments were obtained following intake and initial treatment. Temperament was rated with the 39-item version of the self-rated TEMPS-A scale [36]. We considered raw, average numerical scores for five individual temperaments (cyclothymic [*cyc*], 12 items; dysthymic [*dys*], irritable [*irr*], hyperthymic [*hyp*], 8 items each; and anxious [*anx*], 3 items). Clinical measures were: diagnosis (BD-1, BD-2, MDD) score for the Hamilton Depression Rating Scale (HDRS_21_), any suicidality (ideation or acts), or suicidal acts, substance abuse, episodes/year, and %-time ill overall or in depression or [hypo]mania or total. For multivariate modeling, factors with preliminary bivariate differences were included stepwise as covariates.

### Data analysis

Data are presented as means ± standard deviation or with 95% confidence intervals (CI). Differences in TEMPS-A scores, sociodemographic factors, and morbidity indices were evaluated using contingency tables (*χ*^2^) for categorical variables or analysis of variance (*t*-test) for continuous data, followed by post hoc comparisons, or with bivariate linear regression (*r*) to compare continuous measures. Statistics arising from preliminary bivariate comparisons were used to guide selection of factors to include in multivariable modeling, so as to limit effects of multiple comparisons. Statistics provided in tables are not repeated in the text. Analyses employed commercial software: Statview.5 (SAS Institute, Cary, NC) for spreadsheets, and Stata.13 (StataCorp, College Station, TX) for analyses.

## Results

### Subject characteristics

A total of 858 adults included: BD (*n* = 423), type 1 (*n* = 173) or 2 (*n* = 250), or MDD (*n* = 435). They were treated clinically and followed prospectively and systematically over an average of 7.99 [7.19–8.79] years. Age at intake averaged 46.2 [45.7–46.7] years; 62.9% [61.4–64.3] of subjects were women.

### TEMPS-A subscores versus diagnosis

We compared scores for the five temperament types with three diagnoses (Table 1). Diagnostic subgroups differed highly significantly in TEMPS-A ratings of cyclothymic (*cyc*) and anxious (*anx*) temperament (*p* < 0.001 overall for both). The *cyc* ratings ranked among the diagnoses as: BD-2 > BD-1 > MDD; *anx*, ratings ranked: MDD > BD-2 > BD-1. Also significant were ratings for irritable temperament (*irr*) which ranked: BD-2 ≥ BD-1 > MDD and for dysthymic temperament scores (*dys*), ranking: MDD > BD-2 > BD-1 (*p* = 0.01 overall for both). Ratings of hyperthymia (*hyp*) did not differ significantly among the diagnoses (*p* = 0.10).

**Table 1.** TEMPS-A temperament scores versus diagnosis.

Temperament	Diagnosis (mean score [95%CI])			*t*-Score	*p*-Value
	BD-1 (*n* = 173)	BD-2 (*n* = 250)	MDD (*n* = 435)		
Cyclothymic	5.32 [4.83–5.81]	5.91 [5.51–6.31]	4.80 [4.51–5.09]	31.7	<0.0001^a^
Anxious	1.24 [1.06–1.42]	1.40 [1.25–1.55]	1.64 [1.51–1.77]	2.82	0.0004^b^
Irritable	1.79 [1.52–2.07]	1.88 [1.62–2.13]	1.47 [1.31–1.64]	2.09	0.01^c^
Dysthymic	3.22 [2.91–3.53]	3.56 [3.29–3.83]	3.77 [3.57–3.96]	2.07	0.01^d^
Hyperthymic	3.69 [3.35–4.02]	3.65 [3.39–3.92]	3.35 [3.15–3.55]	1.52	0.10

*Note:* Temperaments are ranked by significance of diagnostic differences. Significant (*p* ≤ 0.05) post hoc comparisons: ^a^BD-2 > BD-1 > MDD; ^b^MDD > BD-2 > BD-1; ^c^BD-2 = BD-1 > MDD; ^d^MDD > BD-2 > BD-1. Scores vary with the number of items for each temperament type (e.g., 12 for cyclothymic and 3 for anxious).

*Abbreviations: BD-1, type I bipolar disorder; BD-2, type II bipolar disorder; MDD, major depressive disorder.*

In addition, the ratio of individual scores for *cyc/anx* strongly differentiated BD (3.89 [3.55–4.23]) from MDD (2.70 [2.48–2.92]) patients (*t* = 5.94, *p* < 0.0001), whereas this ratio was very similar in BD-1 (3.83 [3.28–4.38]) and BD-2 (3.93 [3.50–4.36]) cases. The preceding findings suggest that high ratings for *cyc* and low scores for *anx* may help to differentiate BD from MDD, with additional contributions by high *irr* scores and low *dys* ratings also favoring BD over MDD.

### Clinical features associated with temperament ratings

Among all 858 mood disorder participants, several clinically important features were associated with particular temperament ratings, irrespective of diagnosis (Table 2). Ratings of cyclothymic temperament (*cyc*) were strongly associated with: alcohol abuse and substance abuse of any kind, as well as suicidal ideation or acts and suicidal acts (attempts and suicides) specifically. These scores were also significantly correlated with higher depression ratings at intake (HDRS_21_), a greater proportion of time ill during several years of follow-up, and with a higher recurrence frequency (episodes/year).

**Table 2.** Clinical factors associated with TEMPS-A temperament assessment scores for 858 patients diagnosed with a DSM-5 major mood disorder.

Factor	Mean TEMPS score or slope versus TEMPS score [95% CI]		RR	*t*-Score	*p*-Value
	Factor present	Factor absent			
Cyclothymic					
HDRS_21_ score	Slope = 0.387 [0.228–0.545]		–	4.79	<0.0001
%-Time ill	Slope = 1.01 [0.343–1.68]		–	2.97	0.003
Episodes/year	Slope = 0.090 [0.030–0.150]		–	2.96	0.003
Alcohol abuse	6.58 [5.92–7.23]	5.08 [4.85–5.32]	1.30	4.27	<0.0001
Any suicidality	5.58 [5.39–5.86]	4.63 [4.31–4.96]	1.21	4.22	<0.0001
Suicidal acts	6.12 [5.58–6.66]	5.03 [4.80–5.26]	1.22	3.83	0.0001
Any substance abuse	5.94 [5.49–6.38]	5.03 [4.78–5.27]	1.18	3.50	0.0005
Dysthymic					
%-Time depressed	Slope = 1.55 [0.513–2.58]		–	3.37	0.0008
Any suicidality	3.85 [3.66–4.04]	3.16 [2.95–3.37]	1.22	4.97	<0.0001
Suicidal acts	4.23 [3.87–4.58]	3.46 [3.30–3.61]	1.22	4.03	<0.0001
Irritable					
%-Time [hypo]manic	Slope = 0.763 [0.318–1.21]		–	3.37	0.0008
Episodes/year	Slope = 0.132 [0.029–0.234]		–	2.52	0.01
Depressions/year	Slope = 0.069 [0.0006–0.137]		–	1.98	0.05
Substance abuse	2.36 [2.07–2.65]	1.44 [1.31–1.58]	1.64	6.13	<0.0001
Alcohol abuse	2.52 [2.08–2.96]	1.54 [1.41–1.67]	1.64	4.80	<0.0001
Suicidal acts	2.12 [1.77–2.48]	1.54 [1.41–1.67]	1.38	3.49	0.0005
Hyperthymic					
Any suicidality	3.25 [3.07–3.55]	3.88 [3.65–4.11]	0.838	−4.09	<0.0001
Suicidal acts	3.03 [2.68–3.38]	3.58 [3.48–3.74]	0.846	−2.83	0.005
Anxious					
Substance abuse	1.21 [1.03–1.38]	1.57 [1.48–1.66]	0.771	−3.69	0.0002

*Note:* Clinical factors were assessed during an average of 7.99 [7.19–8.79] years of prospective follow-up. All morbidity indices assessed were: depressions, [hypo]manias, or total episodes per year; % time depressed, manic or total; HDRS_21_ score; any suicidality (acts + ideation), suicidal acts, substance abuse, and alcohol abuse.

Dysthymic temperament ratings (*dys*) were also highly significantly greater with both suicidal acts or any form of suicidality (including ideation and acts), and correlated strongly with higher percentage of time depressed.

Irritable temperament scores (*irr*) were highly significantly greater in patients with co-occurring abuse of any substances or of alcohol specifically, and with suicidal acts, and significantly correlated with more depressions/year, episodes/year, and especially with %-of-time during follow-up in mania or hypomania (“[hypo]mania”).

As expected [14], scores for hyperthymic temperament (*hyp*) were highly significantly *lower* (by 15–16%) in patients with a history of suicidal acts or with any suicidality (ideation or acts).

Ratings of anxious temperament (*anx*) were found to be significantly lower (by 23%) in patients with co-occurring substance abuse than among those without substance abuse (Table 2).

### Diagnostic types associated with temperament ratings

Some of the preceding findings among all mood disorder subjects were found selectively in BD or MDD, or occurred with both diagnoses (Table 3). Higher intake HDRS_21_ scores were strongly associated with higher scores for cyclothymic temperament (*cyc*) in both BD and MDD patients, as well as with any lifetime suicidal ideation or acts, but with suicidal acts only among BD patients. Other clinical factors also were associated with higher *cyc* scores selectively with BD but not MDD, notably including abuse of alcohol or of any substance, as well as %-time-ill and episodes/year.

**Table 3.** Clinical factors associated with TEMPS-A temperament assessment subscores with diagnoses of bipolar disorder versus major depressive disorder.

Factor	Mean TEMPS score or slope versus TEMPS score [95% CI]							
	Bipolar disorder				Major depression			
	Factor present	Factor absent	*t*-Score	*p*-Value	Factor present	Factor absent	*t*-Score	*p*-Value
Cyclothymic								
HDRS_21_ score	Slope = 0.490 [0.254–0.727]		4.01	<0.0001	Slope = 0.349 [0.137–0.561]		3.24	0.001
%-Time ill	Slope = 1.20 [0.323–2.09]		2.69	0.008	Slope = 0.04 [−0.96 to 1.04]		0.07	0.94
Episodes/year	Slope = 0.116 [0.028–0.204]		2.59	0.01	Slope = −0.008 [−0.071 to 0.054]		0.27	0.79
Any substance abuse	6.73 [5.99–7.47]	5.48 [5.13–5.84]	2.95	0.003	6.00 [3.79–8.21]	4.74 [4.44–5.04]	1.80	0.07
Alcohol abuse	6.73 [5.99–7.47]	5.48 [5.13–5.84]	2.95	0.003	6.00 [4.51–7.49]	4.74 [4.44–5.04]	1.80	0.07
Suicidal acts	6.39 [5.72–7.06]	5.45 [5.10–5.80]	2.53	0.01	5.54 [4.60–6.49]	4.66 [4.35–4.97]	1.87	0.06
Any suicidality	5.95 [5.57–6.34]	5.10 [4.59–5.62]	2.52	0.01	5.14 [4.74–5.53]	4.27 [3.70–4.85]	2.92	0.004
Dysthymic								
%-Time depressed	Slope = 1.55 [0.513–2.58]		2.94	0.003	Slope = 1.50 [0.05–2.95]		2.05	0.04
Suicidal acts	4.19 [3.76–4.62]	3.20 [297–3.44.]	4.07	<0.0001	4.30 [3.65–4.96]	3.68 [3.47–3.89]	1.96	0.05
Any suicidality	3.63 [3.37–3.89]	3.05 [2.73–3.37]	2.59	0.01	4.12 [3.86–4.39]	3.24 [2.96–3.53]	4.39	<0.0001
Irritable								
%-Time[hypo]manic	Slope = 0.773 [0.123–1.42]		2.34	0.02	Slope = 0.026 [−0.040 to 0.100]		0.74	0.46
Episodes/year	Slope = 0.103 [−0.042 to 0.249]		1.40	0.16	Slope = 0.107 [−0.006 to 0.220]		1.87	0.06
Depressions/year	Slope = 0.038 [−0.049 to 0.125]		0.85	0.39	Slope = 0.098 [−0.015 to 0.210]		1.72	0.09
Any substance abuse	2.51 [2.15–2.88]	1.58 [1.37–1.80]	4.56	<0.0001	2.02 [1.56–2.48]	1.32 [1.15–1.50]	2.92	0.004
Alcohol abuse	2.63 [2.13–3.14]	1.72 [1.52–1.93]	3.56	0.0004	2.11 [1.16–3.05]	1.38 [1.21–1.55]	1.84	0.07
Suicidal acts	2.27 [1.81–2.72]	1.72 [1.52–1.92]	2.45	0.02	1.80 [1.25–2.36]	1.38 [1.21–1.55]	1.59	0.11
Hyperthymic								
Suicidal acts	3.07 [2.63–3.51]	3.83 [3.59–4.07]]	−3.06	0.002	2.96 [2.37–3.55	3.37 [3.54–3.58]	−1.29	0.20
Any suicidality	3.45 [3.18–3.71]	4.07 [3.72–4.42]	−2.80	0.007	3.04 [2.77–3.30]	3.73 [3.42–4.04]	−3.23	0.0008
Anxious								
Substance abuse	1.11 [0.90–1.31]	1.43 [1.24–1.63]	2.63	0.009	1.43 [1.09–1.77]	1.68 [1.56–1.81]	1.49	0.14

Ratings of dysthymic temperament (*dys*) were associated with %-time-depressed and with suicidal acts or any suicidality in both BD and MDD patients.

Ratings of irritable temperament (*irr*) were elevated with co-occurring substance abuse in both BD and MDD patients. However, the following factors were associated with higher *irr* scores only with BD: alcohol abuse specifically, suicidal acts, and %-time [hypo]manic, whereas depressions/year and mood episodes/year were not significantly associated with *irr* scores in either BD or MDD patients.

Hyperthymic temperament ratings (*hyp*) were selectively lower with suicidal *acts* only among BD, but not MDD patients. However, any suicidality, including suicidal ideation as well as suicidal acts, was associated with lower *hyp* scores in both diagnostic groups.

Lower ratings of anxious temperament (*anx*) were associated with substance abuse only among BD patients and not with MDD (Table 3).

### Multivariable regression modeling

Based on the preliminary findings just summarized, we constructed multivariable logistic regression models for associations of temperament ratings with diagnosis (BD vs. MDD), substance abuse, and suicidal acts. In addition, we used linear regression models for %-time during prospective follow-up in [hypo]mania or depression (Table 4). Diagnosis of BD was more likely than MDD with increased ratings for *cyc*, lower scores for anx and *dys*, and lower ratings for *irr.* In addition, a particularly strong differentiating factor was the ratio of *cyc/anx* ratings. In logistic regression modeling, along with lower *dys* and higher *irr* scores, the ratio of *cyc/anx* scores separated BD from MDD very strongly (OR = 1.22 [1.14–1.32], *χ*^2^ = 28.8, *p* < 0.0001). The proportion of time in [hypo]mania was associated with decreased *anx*, and increased *cyc*, *hyp*, and *irr* scores. In contrast, the %-time in depression was strongly and selectively associated only with higher *dys* scores. Substance abuse was associated strongly with increased *irr* scores, lower *anx* scores, and with BD more than MDD. Finally, suicidal acts (attempts and suicides) were strongly associated with BD > MDD and with higher *dys* ratings, and less strongly with increased *irr* and lower *hyp* scores (Table 4).

**Table 4.** Multivariable models for clinical outcomes.

Factor	OR or Slope (*β*) [95%CI]	*χ*^2^ or *t*-Score	*p*-Value
Diagnosis: BD > MDD^a^			
Higher *cyc*	1.14 [1.09–1.20]	25.4	<0.0001
Lower *anx*	0.775 [0.686–0.875]	16.9	<0.0001
Lower *dys*	0.859 [0.797–0.926]	15.6	<0.0001
Higher *irr*	1.10 [1.01–1.19]	4.97	0.029
%-Time [Hypo]manic^b^			
Lower *anx*	−0.875 [−1.58 to −0.169]	2.43	0.015
Higher *cyc*	0.325 [0.045–0.606]	2.28	0.023
Higher *hyp*	0.442 [0.053–0.831]	2.23	0.026
Higher *irr*	0.517 [0.039–0.995]	2.12	0.034
%-Time depressed^b^			
Higher *dys*	1.49 [0.670–2.31]	3.57	<0.0001
Substance abuse^a^			
Higher *irr*	1.30 [1.20–1.43]	36.5	<0.0001
BD > MDD	2.26 [1.60–3.20]	21.2	<0.0001
Lower *anx*	0.766 [0.662–0.887]	12.7	0.0004
Suicidal acts^a^			
BD > MDD	2.49 [1.70–3.64]	22.0	<0.0001
Higher *dys*	1.14 [1.04–1.25]	7.62	0.006
Lower *hyp*	0.893 [0.816–0.977]	6.13	0.013
Higher *irr*	1.13 [1.02–1.24]	5.94	0.015

*Note: Lower hyp scores with suicidal acts or ideation were found in preliminary bivariate analyses with both BD and MDD subjects, but selectively with BD for suicidal acts (Table* *3* *).*

*Abbreviations: anx, anxious temperament; BD, bipolar disorder; cyc, cyclothymic temperament; dys, dysthymic temperament; hyp, hyperthymic temperament; irr, irritable temperament; MDD, major depressive disorder.*

a Logistic regression modeling (OR).

b Linear regression modeling (slope).

## Discussion

This study involved data collected longitudinally for an average of 7.99 years of prospective observations of 858 unselected, consecutive individuals (a total of 6,855 person-years). We aimed to compare ratings of affective temperaments in patients diagnosed with a DSM-5 major affective disorder. We found several relationships of clinical interest that differentiated individuals with BD versus MDD and provided predictive associations with other clinical features of interest (Tables 3 and 4). This appears to be the first such study with a large sample size and longitudinal design aimed at evaluating the predictive value of affective temperament ratings with diagnostic and morbidity measures in patients diagnosed reliably with DSM-5 BD-1, BD-2, or MDD.

Multivariable logistic regression modeling revealed that higher scores for *cyclothymic* and *irritable* temperaments were independently more likely among BD than MDD patients, whereas *dysthymic* and *anxious* temperament scores were higher in MDD than BD (Table 4). Moreover, the ratio of relatively high ratings for *cyclothymic* and low scores for *anxious* temperament was especially elevated with BD and distinguished BD from MDD.

Such results are consistent with previous studies comparing affective temperaments in patients with major mood disorders, in which BD cases showed higher *cyclothymic* and *hyperthymic* and lower *anxious* temperament scores than did MDD cases [27]. In addition, *cyclothymic* and *hyperthymic* temperament ratings have been reported to be BD-selective [1]. We also found a preliminary association of BD diagnosis with elevated *hyperthymia* ratings (Table 1) that was not sustained in multivariable modeling (Table 4). Also of interest, relatives of BD patients have been reported to have higher *cyclothymia* scores than family members of MDD cases or healthy controls [27].

Consistent with our findings, Morishita et al. [29,30] found that *cyclothymic* and *anxious* temperament scores significantly differentiated the diagnosis of BD from MDD and statistically associated with BD by using multivariable logistic regression modeling. However, those studies also found that higher *hyperthymic* temperament scores differentiated subjects diagnosed with BD-1 versus BD-2, which we did not find (Table 1). The studies by Morishita et al. [29,30] were based on a cross-sectional design and so may have missed some patients considered to have MDD who might later have met diagnostic criteria for BD [5,37,38]. Moreover, not all of their patients were currently in remission or euthymic, and some responses to temperament categorizing questions may have been influenced by current mood states [34,39].

Our findings confirmed associations of suicidal risk with higher scores of all temperament types except for *hyperthymic*, which were lower (Tables 3 and 4), as had been noted previously [14,40,41]. We also found that higher *cyclothymic* and *irritable* scores and lower *anxious* scores were associated with substance abuse. Though few previous studies focused on the temperament profile in BD patients with abuse of alcohol or other substances, in line with our findings (Table 3), *cyclothymic* or *irritable* temperament was reported to be associated with substance abuse (especially among BD patients) [42]. Low scores of *anxious* with high scores of *irritable* may reflect impulsivity commonly present with substance abuse. In addition, *hyperthymia* was associated with more severe hypomanic symptoms in multivariable modeling (Table 4), and in a previous study of 112 young adults at-risk for BD [42]. *Cyclothymic* and *hyperthymic* traits preceded abuse of stimulants by years, based on evaluating longitudinal progression of the dual pathology in a small sample of BD patients [43]. Among 1420 BD patients, several TEMPS-A scores were higher with alcohol abuse, particularly *irritable* and *hyperthymic* ratings, adjusted for potential confounders [19]. Finally, regression modeling based on 1,090 BD patients found abuse of alcohol and of other substances to be associated with *irritable* and *hyperthymic* temperaments, especially in males [21], whereas we found elevated scores of *cyclothymic* and *irritable* to be selectively associated with abuse of alcohol (Table 2).

We also found that several TEMPS-A scores were higher with measures of greater affective morbidity. In particular, higher ratings for *dysthymia* correlated strongly with the proportion of time in depression with both BD and MDD patients, whereas higher *irritable* ratings significantly correlated with more episodes/year and depressions/year, as well as with the proportion of time of BD subjects in mania or hypomania (“[hypo]mania”; Table 2). Finally, in addition to a strong association between *cyclothymic* temperament and initial depression severity assessed by HDRS_21_ (Table 2) in both MDD and BD subjects (Table 3), higher *cyclothymia* scores correlated significantly with %-time-ill and episodes/year but selectively only among BD subjects (Table 3).

High *cyclothymia* scores seem to be associated with relatively unfavorable prognosis, perhaps as reflecting emotional and behavioral instability. This view is consistent with previous studies’ finding that cyclothymic temperament can affect illness-course adversely [44–46].

Consistent with cyclothymic tendencies, mood reactivity and emotional dysregulation represent core psychopathological dimensions that often develop early in childhood [47]. BD patients, including those with cyclothymic temperament, are often initially misdiagnosed, typically as having MDD, potentially resulting in prolonged delay of appropriate treatment, higher rates of psychiatric comorbidity, and more histrionic, passive–aggressive, and less obsessive–compulsive personality types compared to those affected by BD without *cyclothymic* temperament—all probably tending to limit chances of attaining clinical remission [47,48].

In a sample of 51 remitted BD subjects followed for 24 months, *cyclothymic* temperament scores were associated with greater overall functional impairment, including home management, and both individual and social leisure activities [45]. In line with the present study, with respect to BD patients (Table 3), high *cyclothymia* ratings have been reported to predict an excess of affective recurrences even when controlling for medication nonadherence [45]. Finally, *cyclothymic* temperament in BD patients has been associated with inferior treatment adherence and inferior response to medication [20,49] as well as to psychoeducation [50].

### Limitations

Some individuals may not have been fully euthymic, as their TEMPS-A assessments occurred early in their clinical assessment, soon after clinic entry, but none was acutely ill. Efforts to limit effects of multiple comparisons include the recommendation to consider of particular interest initial bivariate comparisons yielding *p*-value of ≤0.01, and the limited statistics arising from multivariable modeling.

## Conclusions

The present findings support the clinical value of rating affective temperament types, including to help differentiate BD from MDD diagnoses, limit risk of later changing diagnosis from MDD to BD, and to predict morbidity (mood states, recurrence rates, substance abuse, and suicidal risk), as well as highlighting the value of *cyclothymic* mood instability as a generally adverse prognostic indicator. The findings presented require replication and extension. Assessment of temperament is easily attained with the TEMPS-A scale, and should be considered as a component of routine evaluation of mood disorder patients, with possible particular value in the often difficult task of predicting a change of diagnosis from MDD to BD.

## Data Availability

The data that support the findings of this study are available from L.T. Restrictions are applied, given confidentiality issues.
